# Supplementary material for: Predicting bacterial promoter function and evolution from random sequences
Source: eLife. 2022 Jan 26;11:e64543. doi: 10.7554/eLife.64543 (PMC8791639; doi:10.7554/eLife.64543)
Supplement: Figure 1—source data 1. — For each position in the energy matrix, the energy penalty is normalized to the lowest energy (strongest binding) residue in that position, which is set to zero. Same applies for the spacer variation penalty, where the optimal spacer length is set to zero. Orange background marks the canonical –10 and –35 binding sites of σ70-RNAP. For pairwise interaction, ‘identity’ refers to the two positions and specific residues that have a significant positive (destabilizing) or negative (stabilizing) interaction, with numbers indicating the position of the residue in the matrix shown in Figure 1C. Shaded cells indicate the strongest interactions, which are shown in Figure 1C. The numbers indicate the position in the energy matrix, starting from the most upstream to the most downstream (left to right in the energy matrix shown in Figure 1C). [file elife-64543-fig1-data1.zip › Figure 1 Source Data 1.docx]

**Figure 1 – Source Data 1. Inferred values for model parameters**. For each position in the energy matrix, the energy penalty is normalized to the lowest energy (strongest binding) residue in that position, which is set to zero. Same applies for the spacer variation penalty, where the optimal spacer length is set to zero. Orange background marks the canonical -10 and -35 binding sites of σ^70^-RNAP. For Pairwise Interaction, **‘**identity’ refers to the two positions and specific residues that have a significant positive (destabilizing) or negative (stabilizing) interaction, with numbers indicating the position of the residue in the matrix shown in Fig.1C. Shaded cells indicate the strongest interactions, which are shown in Fig.1C. The numbers indicate the position in the energy matrix, starting from the most upstream to the most downstream (left to right in the energy matrix shown in Fig.1C).

|  | Residue | A | C | G | T |  |
| --- | --- | --- | --- | --- | --- | --- |
|  | most upstream position | 1.0042 | 0.8725 | 0.9656 | 0 |  |
|  |  | 0 | 0.7132 | 0.2516 | 1.4211 |  |
|  |  | 1.4277 | 1.5981 | 0.9806 | 0 |  |
|  |  | 0.8669 | 0.9585 | 0 | 0.4791 |  |
|  |  | 0.673 | 0.3131 | 0 | 0.3544 |  |
| **Energy Matrix** |  | 4.8851 | 3.7094 | 4.566 | 0 |  |
| **-10 foot** |  | 0 | 5.8406 | 6.3724 | 4.2602 |  |
|  |  | 1.1993 | 1.3188 | 1.5041 | 0 |  |
|  |  | 0 | 1.9175 | 2.0824 | 1.7399 |  |
|  |  | 0 | 0.8898 | 2.1099 | 2.3318 |  |
|  |  | 4.2699 | 4.646 | 4.8871 | 0 |  |
|  | most downstream position | 0 | 0.8024 | 0.3061 | 0.3178 |  |
|  |  |  |  |  |  |  |
|  | most upstream | 0 | 0.6782 | 0.2726 | 0.1205 |  |
|  |  | 0.3426 | 0 | 0.2543 | 2.0091 |  |
|  |  | 4.1376 | 4.9489 | 3.7397 | 0 |  |
|  |  | 2.2569 | 3.995 | 2.4141 | 0 |  |
|  |  | 5.4228 | 4.5391 | 0 | 2.2583 |  |
| **Energy Matrix** |  | 0 | 1.621 | 3.9457 | 2.3814 |  |
| **-35 foot** |  | 1.7288 | 0 | 2.1718 | 1.5128 |  |
|  |  | 0 | 1.3907 | 1.1822 | 0.5051 |  |
|  |  | 0.4337 | 0.7953 | 0.9523 | 0 |  |
|  |  | 0.0977 | 0.4765 | 0.5841 | 0 |  |
|  |  | 0.2763 | 0.1117 | 0.4113 | 0 |  |
|  | most downstream | 0.0844 | 0.1982 | 0 | 0.0121 |  |
|  |  |  |  |  |  |  |
| **Spacer** | difference to optimal (9bp) spacer length | -2 | -1 | 0 | 1 | 2 |
|  | energy penalty | 8.514 | 2.122 | 0 | 1.242 | 5.346 |
|  |  |  |  |  |  |  |
| **Clearance rate** |  | 0.332 |  |  |  |  |
|  |  |  |  |  |  |  |
| **Chemical potential** | library | *P_R_ / P_L_* | *36N* |  |  |  |
|  |  | 11.128 | 10.531 |  |  |  |

**Pairwise interactions**

| **Identity** | **Interaction strength** |
| --- | --- |
| (2G, 24A) | -0.076 |
| (9A, 10A) | -0.083 |
| (8T, 9T) | -0.091 |
| (23T, 24G) | -0.092 |
| (22T, 26G) | -0.094 |
| (24A, 25T) | -0.094 |
| (24A, 26G) | -0.101 |
| (27T, 28A) | -0.212 |
| (6C, 7A) | -0.238 |
| (5C, 6C) | -0.275 |
| (6T, 7G) | -0.308 |
| (4C, 9C) | -0.342 |
| (6A, 7A) | 0.058 |
| (23G, 29C) | 0.062 |
| (6A, 32A) | 0.092 |
| (25G, 26G) | 0.106 |
| (6C, 7C) | 0.109 |
| (23T, 25T) | 0.123 |
| (23T, 26G) | 0.127 |
| (2A, 24A) | 0.128 |
| (24A, 26T) | 0.135 |
| (23A, 28G) | 0.150 |
| (6A, 7G) | 0.155 |
| (22A, 23A) | 0.168 |
| (22A, 24C) | 0.170 |
| (2A, 27T) | 0.179 |
| (23T, 24C) | 0.188 |
| (1G, 27T) | 0.243 |
| (25A, 27T) | 0.257 |
| (23A, 27T) | 0.329 |
| (23T, 24T) | 0.390 |
