# Supplementary material for: Predicting bacterial promoter function and evolution from random sequences
Source: eLife. 2022 Jan 26;11:e64543. doi: 10.7554/eLife.64543 (PMC8791639; doi:10.7554/eLife.64543)
Supplement: Figure 3—source data 1. — The table shows the number of reads remaining in the datasets following each step of data processing, from original sequenced library down to the final library used for model fitting and evaluation. [file elife-64543-fig3-data1.zip › Figure 3 Source Data 1.docx]

**Figure 3 – Source Data 1. Processing of the mutant libraries and sizes of datasets after splits**. The table shows the number of reads remaining in the datasets following each step of data processing, from original sequenced library down to the final library used for model fitting and evaluation.

|  | ***P_R_*** | ***P_L_*** |
| --- | --- | --- |
| **Initial number of reads** | 7,138,685 | 9,149,460 |
| **Filtered on 0 mismatches** | 5,432,101 | 6,557,151 |
| **Condition on same (and valid) left and right bin tags** | 2,637,166 | 2,459,553 |
| **Number of unique sequences** | 335,060 | 324,558 |
| **Condition on length being within 4bp of canonical** | 329,672 | 318,946 |
| **Condition on coverage of at least 10** | 29,045 | 6,710 |
| **Cond. on Shine-Dalgarno within +/- 5bp of canonical** | 29,031 | 6,694 |
| **Remove sequences too different from ancestor** | 29,020 | 6,415 |
| **Remove sequences with expression st.dev. > 0.5** | 22,884 | 4,239 |
| **Condition on median, mode and mean of expression**  **distribution being within 0.5** | 22,769 | 4,222 |
| **Condition on coverage of at least 30** | 12,476 | 2,984 |

|  | ***36N*** |
| --- | --- |
| **Initial number of reads** | 10,124,219 |
| **Filtered on 0 mismatches** | 9,917,488 |
| **Discard (yet save) reads that map to control sequence** | 8,772,436 |
| **Discard reads that cannot map left and right flanking region** | 7,031,460 |
| **Condition on the length of the core region within 2bp of canonical** | 6,498,273 |
| **Number of unique sequences** | 90,071 |
| **Condition on coverage of at least 2 (temporarily)** | 24,527 |
| **Condition on coverage of at least 30** | 13,341 |
