# Supplementary material for: Predicting bacterial promoter function and evolution from random sequences
Source: eLife. 2022 Jan 26;11:e64543. doi: 10.7554/eLife.64543 (PMC8791639; doi:10.7554/eLife.64543)
Supplement: Figure 3—source data 2. — Bins are no (‘0’), low (‘1’), intermediate (‘2’), and high (‘3’) for the PR and PL libraries, and are ordered from lowest (‘0’) to highest (‘11’) for the 36N library. [file elife-64543-fig3-data2.zip › Figure 3 Source Data 2.docx]

**Figure 3 – Source Data 2. Number of mutants per expression bin for each split of the P_R_, P_L_, and *36N* dataset**. Bins are no (‘0’), low (‘1’), intermediate (‘2’) and high (‘3’) for the P_R_ and P_L_ libraries , and are ordered from lowest (‘0’) to highest (‘11’) for the *36N* library.

**Sizes of datasets after the splits**

|  | **Training** | **Validation** | **Evaluation** | **Total** |
| --- | --- | --- | --- | --- |
| ***P_R_*** | 7,485 | 2,495 | 2,496 | 12,476 |
| ***P_L_*** | 1,790 | 597 | 597 | 2,984 |
| ***36N*** | 8,004 | 2,668 | 2,669 | 13,341 |

**Number of mutants per bin**

| ***P_R_*** | **0** | **1** | **2** | **3** |
| --- | --- | --- | --- | --- |
| **evaluation** | 276 | 847 | 450 | 923 |
| **validation** | 290 | 848 | 437 | 920 |
| **training** | 831 | 2,363 | 1,375 | 2,916 |

| ***P_L_*** | **0** | **1** | **2** | **3** |
| --- | --- | --- | --- | --- |
| **evaluation** | 152 | 144 | 88 | 213 |
| **validation** | 173 | 130 | 90 | 204 |
| **training** | 450 | 399 | 246 | 695 |

| ***36N*** | **0** | **1** | **2** | **3** | **4** | **5** | **6** | **7** | **8** | **9** | **10** | **11** |
| --- | --- | --- | --- | --- | --- | --- | --- | --- | --- | --- | --- | --- |
| **evaluation** | 177 | 1,477 | 424 | 220 | 107 | 77 | 87 | 45 | 26 | 16 | 7 | 6 |
| **validation** | 164 | 1,509 | 453 | 226 | 105 | 65 | 70 | 42 | 15 | 11 | 7 | 1 |
| **training** | 483 | 4,535 | 1,296 | 648 | 345 | 216 | 205 | 124 | 72 | 33 | 22 | 25 |
